# Supplementary material for: Learning curve and surgical outcome of robotic assisted colorectal surgery with ERAS program
Source: Sci Rep. 2022 Nov 29;12:20566. doi: 10.1038/s41598-022-24665-w (PMC9709162; doi:10.1038/s41598-022-24665-w)
Supplement: Supplementary file 2 — Supplementary Information 2. [file 41598_2022_24665_MOESM2_ESM.zip › RAL Raw data-Ñ[▒K/118/Patho_ñw╝╨░O▒Kñσ.pdf]

臺中榮民總醫院 臺中市臺灣大道四段1650號 病理部活體組織切片 臨床醫學實驗及病理診斷中心 檢驗(查)報告

[redacted]

Pathologic diagnosis:
1. Rectum, robotic low anterior resection --- Adenocarcinoma, moderately differentiated. Tumor invaded to mesocolic soft tissue with subserosal chronic follicular inflammation. The bilateral cut ends and mesocolic lymph nodes (0/19) are free of tumor.
2. IMA root, dissection --- Unremarkable fibrovascular tissue and lymph nodes (0/6).
3. Rectum, cut end, distal, resection --- Uninvolved by carcinoma.
4. Omentum, resection --- Mild congestion. No tumor seen.

Ancillary study for diagnosis:
IHC stains for PMS2 and MSH6 are done on section D. All above markers are preserved.

Prognostic and predictive factor:
1. Tumor size: 4.5x2.5 cm.
2. Depth of invasion: Pericolorectal tissue.
3. Histologic grade: G2: Moderately differentiated.
4. Angiolymphatic invasion: Not identified.
5. Perineural invasion: Present.
6. Tumor deposits (discontinuous extramural extension): Not identified.
7. Macroscopic tumor perforation: Not identified.
8. Surgical Margins: a) Proximal Margin: Uninvolved by invasive carcinoma, distance of tumor from margin: 11 cm. b) Distal Margin: Uninvolved by invasive carcinoma, distance of tumor from margin: 6.5 cm. c) Radial or Mesenteric Margin: Uninvolved by invasive carcinoma, distance of tumor from margin: 4 mm.
9. Lymph node status: Uninvolved (0/19).
10. Extranodal involvement: Not identified.
11. Tumor regression grading S/P CCRT: No known presurgical therapy.
12. Type of polyp in which invasive carcinoma arose: Not identified.
13. Additional pathologic findings: Not identified.
14. Pathological TNM Stage: pT3N0(According to the eighth edition, American Joint Committee on Cancer Staging Guidelines for Tumors).
15. TNM descriptors: Not applicable.

Gross description:
The specimen consists of 1) a segment of colon, 22 cm in length and 4 cm in proximal cut end circumference, 6 cm in distal circumference, and perirectal soft tissue. A gray tan firm annular elevated and centrally ulcerative tumor, 4.5x2.5 cm, is noted, 6.5 cm to the distal cut end. On cross section, the tumor invades to the mesocolic soft tissue with 4 mm to the radial margin. The both cut ends are free of tumor grossly. 2) IMA root, a piece of tan yellow soft tissue, 3.5x2.1x0.6 cm. 3) distal cut end, a piece of tan mucosa-covered soft tissue, 2x1x0.5 cm. 4) omentum, a sheet of yellow soft tissue, 15x13x0.8 cm. Representative part for section: A) proximal cut end B) distal cut end C-F) tumor G-J) lymph nodes K-L) specimen 2 M) specimen 3 N-P) specimen 4.

Note:
1. The interpretation of MMR panel and recommended studies are as below: (1) No loss of nuclear expression of MMR proteins: low probability of microsatellite instability-high (MSI-H) . (2) Loss of nuclear expression of MLH1 and PMS2: testing for methylation of the MLH1 promoter and/or mutation of BRAF is indicated (the presence of a BRAF V600E mutation and/or MLH1 methylation suggests that the tumor is sporadic and germline evaluation is probably not indicated; absence of both MLH1 methylation and of BRAF V600E mutation suggests the possibility of Lynch syndrome, and sequencing and/or large deletion/duplication testing of germline MLH1 may be indicated). (3) Loss of nuclear expression of MSH2 and MSH6: high probability of Lynch syndrome (sequencing and/or large deletion/duplication testing of germline MSH2 may be indicated, and, if negative, sequencing and/or large deletion/duplication testing of germline MSH6 may be indicated). (4) Loss of nuclear expression of MSH6 only: high probability of Lynch syndrome (sequencing and/or large deletion/duplication testing of germline MSH6 may be indicated). (5) Loss of nuclear expression of PMS2 only: high probability of Lynch syndrome (sequencing and/or large deletion/duplication testing of germline PMS2 may be indicated). (6) Please note: There are exceptions to the above IHC interpretations. These results should not be considered in isolation, and clinical correlation with genetic counseling is recommended to assess the need for germline testing.

2. This case has been peer reviewed by two doctors.
#T-59600_2 #M-81403_2 2 1105 S0000F
#T-C4560_2 #M-09183_2 0 1105 000000

已電子簽章

警告：本單僅供醫師參考使用，不具任何申請效力！！
請病患妥為保管，若不慎遺失將自負個人資料外洩責任！
